# Prevalence, genotyping and risk factors of thermophilic *Campylobacter* spreading in organic turkey farms in Germany

**DOI:** 10.1186/s13099-016-0108-2

**Published:** 2016-06-02

**Authors:** Marwa Fawzy El Metwaly Ahmed, Hosny El-Adawy, Helmut Hotzel, Herbert Tomaso, Heinrich Neubauer, Nicole Kemper, Joerg Hartung, Hafez Mohamed Hafez

**Affiliations:** Institute for Animal Hygiene, Animal Welfare and Farm Animal Behaviour, University of Veterinary Medicine Hannover, Foundation, Hannover, Germany; Institute of Bacterial Infections and Zoonoses, Friedrich-Loeffler-Institut, Jena, Germany; Institute for Poultry Diseases, Free University Berlin, Berlin, Germany; Department of Hygiene and Zoonoses, Faculty of Veterinary Medicine, Mansoura University, Mansoura, Egypt; Department of Poultry Diseases, Faculty of Veterinary Medicine, Kafrelsheikh University, Kafr El-Sheikh, Egypt

**Keywords:** Thermophilic *Campylobacter*, Organic turkey, Genotyping, Water, Beetles

## Abstract

**Background:**

The need for organic food of animal origin has increased rapidly in recent years. However, effects of organic animal husbandry on food safety have not been rigorously tested especially in meat turkey flocks. This study provides for the first time an overview on the prevalence and genetic diversity of *Campylobacter* species (spp.) in five organic meat turkey farms located in different regions in Germany, as well as on potential risk factors of bacterial spreading. Thirty cloacal swabs as well as water samples and darkling beetles were collected from each flock and examined for the presence of *Campylobacter* by conventional and molecular biological methods. The isolates were genotyped by *fla*A-RFLP.

**Results:**

*Campylobacter* spp. were detected in cloacal swabs in all 5 turkey flocks with prevalence ranged from 90.0 to 100 %. 13 cloacal swabs collected from birds in farm III and IV were harboured mixed population of thermophilic campylobacters. In total, from 158 *Campylobacter* isolated from turkeys 89 (56.33 %) were identified as *C. coli* and 69 (43.76 %) as *C. jejuni*. Three *Campylobacter* (2 *C. jejuni* and 1 *C. coli*) were detected in drinkers of two farms and 3 *C. coli* were isolated from darkling beetles of one farm. No *Campylobacter* were isolated from main water tanks. *fla*A-RFLP assay showed that turkey farms can harbour more than one genotype. In a single turkey two different genotypes could be detected. The genotypes of campylobacters isolated from water samples or beetles were identical with those isolated from turkeys. No effect was found of some environmental parameters [ammonia concentration (NH_3_), carbon dioxide concentration (CO_2_), relative humidity (RH) and air temperature)] on *Campylobacter* prevalence in organic turkey farms. Additionally, drinking water and darkling beetles might be considered as risk factors for the spreading of *Campylobacter* in turkey flocks.

**Conclusions:**

This study highlights the high prevalence and genotypic diversity of *Campylobacter* spp. isolated from organic turkey flocks. Further research is needed to assess other potential risk factors responsible for bacteria spreading in order to mitigate the spread of *Campylobacter* in organic turkey flocks by improving biosecurity control measures.

**Electronic supplementary material:**

The online version of this article (doi:10.1186/s13099-016-0108-2) contains supplementary material, which is available to authorized users.

## Background

Over the last three decades, *Campylobacter* spp. have represented an increasing concern worldwide and appear to be the most common foodborne disease in which, consumption of poultry meat is considered as major, if not largest source of infection [1]. On the other hand, organic livestock farming has grown rapidly and the demand for organic meat consumption has increased substantially. This consideration is mainly supported by consumers’ perception of organic products as healthier and safer [2].

However, on organic farms the microbial safety risk is higher due to more contact with the environment than on conventional farms through the access of the birds to an outdoor run and contact with soil, wild birds and other animals and or their faeces [3, 4].

The pathways by which poultry flocks acquire *Campylobacter* are not yet fully understood in detail. The same applies for the formation of the high genetic diversity of *Campylobacter* which was observed in infected poultry flocks of different ages [5–7]. Horizontal transmission is generally considered to be the most significant mode of *Campylobacter* earning by poultry flocks [8–10]. However, the presence of a specific genotype in the environment of the birds does not in itself prove that also the birds are infected [11]. Assumed risk factors and vectors involved in the spreading are beside wild birds and their faeces insects such as darkling beetles and drinking water. Several studies have shown that beetles were only *Campylobacter* positive when the herd was positive, too [10, 12]. Darkling beetles can play a role in the entry of *Campylobacter* into a broiler flock [13, 14]. Drinking water can be an important vehicle for *Campylobacter* spp. transmission to the entire flock [15–17].

Most experiences with *Campylobacter* in organic poultry production are available from free range laying hens indicating that the access to outdoor scratching areas increases the risk of birds infection [18–21]. Although the organic flocks have access to outdoor areas, the prevalence of *Campylobacter* in organic and conventional broiler farms was found identical [19]. While another study in organic turkey flocks demonstrated a higher prevalence than conventional turkey flocks [22].

The prevalence, risk factors for spreading and genetic diversity of *Campylobacter* in organic turkey production received less attention and to best of our knowledge, no previous research was performed in Germany on *Campylobacter* in organic turkeys at farm level. Therefore, the aim of this study was to assess thermophilic *Campylobacter* prevalence and their genetic diversity in turkeys reared under organic conditions and to estimate the role of water and darkling beetles as potential risk factors for transmission of *Campylobacter* spp. in organic turkey flocks in Germany.

## Methods

### Turkey flocks

Samples were collected from five different organic turkey farms during spring and summer seasons. The farms were located in the north-eastern and western regions of Germany situated in typical rural areas surrounded by arable land. Criteria for barn selection were a usual commercial stock size, and a minimal distance of 1 km to the next livestock. The flock sizes ranged from 1000 to 2000 birds (Kelly BBB or B.U.T. 6) per flock (Table 1).

**Table 1:** Farm description, environmental parameters, water supply sources and system, prevalence and genotyping of *Campylobacter* spp. isolated from organic turkey flocks

	Flock I	Flock II	Flock III	Flock IV	Flock V
Total number of birds/flock	1003	2000	1400	1100	1500
Age of birds (weeks)	8	8	4	8	6
Turkey-line	Kelly BBB	B.U.T. 6	B.U.T. 6	Kelly BBB	B.U.T. 6
Water supply type	Tap water	Tap water	Well water	Tap water	Tap water
Type of drinkers	Cups + pendulous	Cups	Cups + drinking trough	Pendulous	Cups
Number of examined cloacal swabs	30	30	30	30	30
No. of positive cloacal swabs	30	27	30^a^	30^a^	30
Flock prevalence (%)	100	90	100	100	100
No. of isolated *C. jejuni*	8	19	5	17	20
No. of isolated *C. coli*	22	8	30	19	10
Prevalence of *C. jejuni* (%)	26.67	70.37	14.29	47.22	66.67
Prevalence of *C. coli* (%)	73.33	29.63	85.71	52.78	33.33
No. of positive water samples	1 (*C. coli*)	2 (*C. jejuni*)	0	0	0
No. of positive beetles sample	0	3 (*C. coli*)	0	0	0
No. of *C. jejuni* genotypes	3	4	1	2	5
No. of *C*. *coli* genotypes	2	1	2	4	4
Temperature (°C)	15.8	23.1	21.30	21.80	15.4
Relative humidity (RH in %)	63.6	64.2	74.8	58.3	56.6
Ammonia (ppm)	1	14	22	1	0
CO_2_ (ppm)	500	1400	800	350	400

^a^From the same cloacal swabs both *C. coli* as well as *C. jejuni* were isolated in 5 swabs of farm III and 8 swabs of farm IV

### Ethical statement

This study was carried out in strict accordance with the recommendations in the Guide for the Care and Use of Laboratory Animals of the University of Veterinary Medicine Hannover. The protocol (sampling of cloacal swabs from turkeys on farms) was approved by the Animal Welfare Officer of the University.

### Isolation of *Campylobacter*

Isolation was performed in accordance with the ISO 10272-1 (2006) guideline [23].

### Cloacal swabs

In order to estimate the prevalence of *Campylobacter* within the turkey flock, 30 cloacal swabs were taken from randomly selected birds (EUROTUBO^®^, DELTALAB, Spain). The sample size calculation was based on the assumption that the within flock prevalence in *Campylobacter* positive flocks would be 95 %. Samples were transported to the laboratory under cooled conditions for further laboratory investigations. Swabs were streaked directly on the farm onto modified Charcoal Cefoperazone Desoxycholate Agar (mCCDA, Oxoid, Wesel, Germany). Thereafter, each swab was placed in a tube with 9 ml Bolton Broth (Oxoid). Plates and tubes were incubated microaerobically for 4 h at 37 °C then transferred to 42 °C for 42 h. Thereafter, a loopfull from the broth was streaked onto mCCDA and further incubated.

The prevalence of *Campylobacter* within the flock was estimated by the ratio of *Campylobacter* positive birds to the total number of tested birds [24].

### Drinking water

At each farm, around 3 l water samples were collected directly from the main water tank using sterile 500 ml bottles (water samples were collected from 10 cm under the water surface). Additionally, 3 l pooled water samples were taken from the drinkers in the poultry house. All water sampling bottles were contained 10 mg of sodium thiosulfate (0.1 mg per ml water) to neutralize any residual chlorine in the water.

Isolation of *Campylobacter* from water samples was performed using a membrane filtration technique (MFT) according to the method described by Mathewson et al. [25]. Isolation was done using two different volumes of collected water samples (one with 500 ml and other with 1 l). Samples were individually filtered through 0.45 μm sterile cellulose acetate membrane filters (Sartorius AG, Goettingen, Germany). The filter from each duplicate was inserted into tubes filled with 9 ml Bolton broth and other filter was placed on the surface of mCCDA. Plates and tubes were incubated as described above.

### Darkling beetles (*Alphitobius diaperinus*)

Beetles were collected from 10 different places distributed inside the barns (corners and under the drinkers) by turnings over the litter with sterile small shovels. Collected beetles were placed in a sterile plastic container with perforated cover.

In the laboratory only beetles identified as *Alphitobius diaperinus* [26] were analyzed and divided into 5 pools each containing 10 beetles and then aseptically crushed using a sterile mortar. Swabs from the crushed beetles were streaked directly on mCCDA, and then the crushed insects were aseptically transferred into 9 ml Bolton broth and handled as described above.

### Identification of *Campylobacter*

*Campylobacter*-like colonies were obtained by cultivation on Columbia blood agar (Oxoid) and then phenotypically identified [24] including motility testing with phase contrast microscopy and catalase as well as oxidase reactions. Thereafter, initially positive isolates were further identified using the biochemical reaction profiles obtained by the API Campy System (BioMerieux, Germany) according to the instructions of the manufacturer.

### DNA extraction

Genomic DNA was extracted from a 48 h bacterial culture on blood agar plates using High Pure PCR Template Preparation Kits (Roche Diagnostics GmbH, Mannheim, Germany) according to the manufacturer’s instructions. The DNA was eluted in 200 µl elution buffer. DNA was quantified spectrophotometrically using a Nanodrop^®^ ND-1000 (Fisher Scientific GmbH, Schwerte, Germany).

### Species confirmation and *fla*A-RFLP assays

The isolates were confirmed as *C. jejuni* or *C. coli* by using a multiplex PCR (mPCR) assay [17]. *fla*A-restriction fragment length polymorphism (RFLP) analysis was done as previously described [6]. The *fla*A amplicon was digested for 18 h at 37 °C with *Dde*I (Roche Diagnostics GmbH). The DNA segments were separated using 2.5 % agarose gels (Starlab GmbH, Hamburg, Germany) in tris-borate-EDTA buffer at 200 V for 1 h, stained with ethidium bromide and visualized under UV light. Documentation was done using a Bio Imaging System (Syngene, Cambridge, UK).

### Measurements of environmental parameters

The following parameters were measured during the samplings near the bird level (between 9:00 and 11:00 a.m.). Temperature and relative humidity (RH) were measured with a thermo-hygrometer (Rotronic Date logger Hydrolog-D HygroClipSTemperatur/RH (Rotronic GmbH, Ettlingen, Germany) for about 30 min. The spot measurements of ammonia (NH_3_) and carbon dioxide (CO_2_) were carried out once during the samplings using Draegeraccuro^®^ tube pump (Drägerwerk AG & Co. KGaA, Germany) and short term Draeger tube (Drägerwerk AG & Co. KGaA, Germany) number CH20501 for ammonia 5/a and 81 01 811 for carbon dioxide 100/a (Additional files 1, 2).

## Results

*Campylobacter* spp. were isolated from cloacal swabs of all investigated 5 organic turkey flocks. The cloacal swabs collected from 150 birds revealed that 147 birds were identified as *Campylobacter* positive (Table 1). In 13 cloacal swabs collected from birds in farm III and IV, each swab harboured two types of thermophilic campylobacters. In total, from 158 *Campylobacter* isolated from five turkey flocks, 89 (56.3 %) isolates were identified as *C. coli* and 69 (43.7 %) as *C. jejuni*. In total three *Campylobacter* isolate, one *C. coli* and two *C. jejuni* were isolated from the water sample in farm I and II, respectively. Additionally, 3 *C. coli* were isolated from darkling beetles collected from farm II (Table 1).

### Prevalence of *Campylobacter* isolated from turkey

The prevalence of *Campylobacter* was high in all 5 organic turkey farms and ranged from (90 %) in farm II to (100 %) in the other four farms (Table 1). The distribution of *Campylobacter* spp. varied in the different farms. *C. coli* was the most prominent species in three farms (I, III and IV) with shares of 73.33, 85.71 and 52.78 %, respectively. *C. jejuni* isolates dominated in farms II and V with prevalence of 63.33 and 66.67 %, respectively.

From 13 cloacal swabs (5 swabs from farm III and 8 from farm IV), 2 *Campylobacter* isolates were isolated from the same swab.

### Occurrence of *Campylobacter* in water and darkling beetles samples

No *Campylobacter* spp. were detected in the water from the main tank neither with nor without enrichment (Table 1). In the water from drinkers only in farm II *C. jejuni* was found in 500 and 1000 ml after enrichment. In addition, *C. coli* could be also detected in 1000 ml drinker water after enrichment in farm I. From darkling beetles only *C. coli* was isolated from 3 out of 5 pools after enrichment in farm II.

### *fla*A typing of isolated *Campylobacter*

**Fig. 1:**
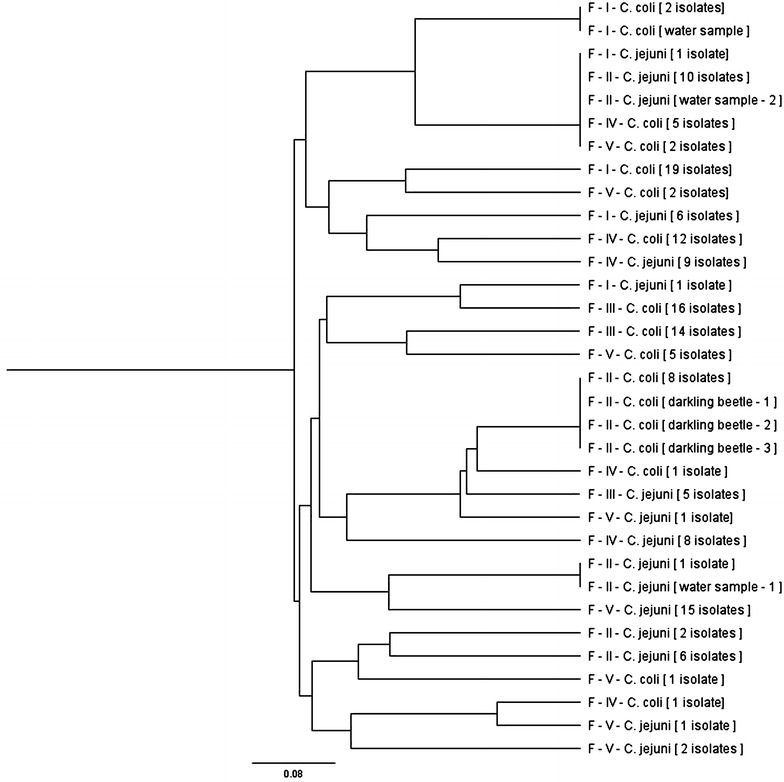
Dendrogram based on restriction profiles of *fla*A gene digested with *Dde*I of 163 *Campylobacter* isolates from 5 turkey farms (FI–FV—farm 1–5)

**Fig. 2:**
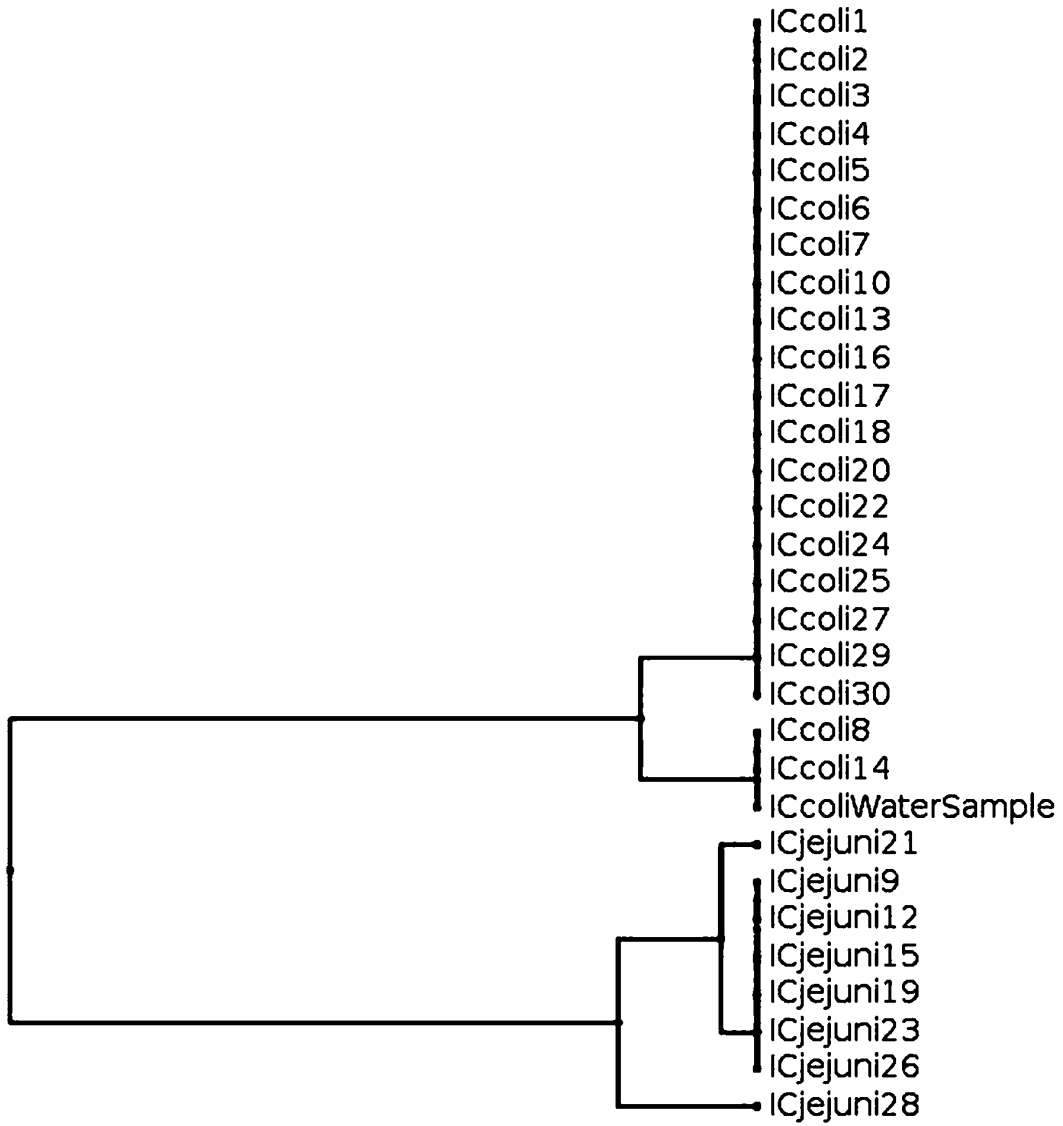
Dendrogram based on restriction profiles of *fla*A gene of 8 *C. jejuni* (7 from cloacal swabs and one from drinking water) and 22 *C. coli* (from cloacal swabs) isolated from farm I (isolate ICcoli21 could not be processed as it was mixed culture)

**Fig. 3:**
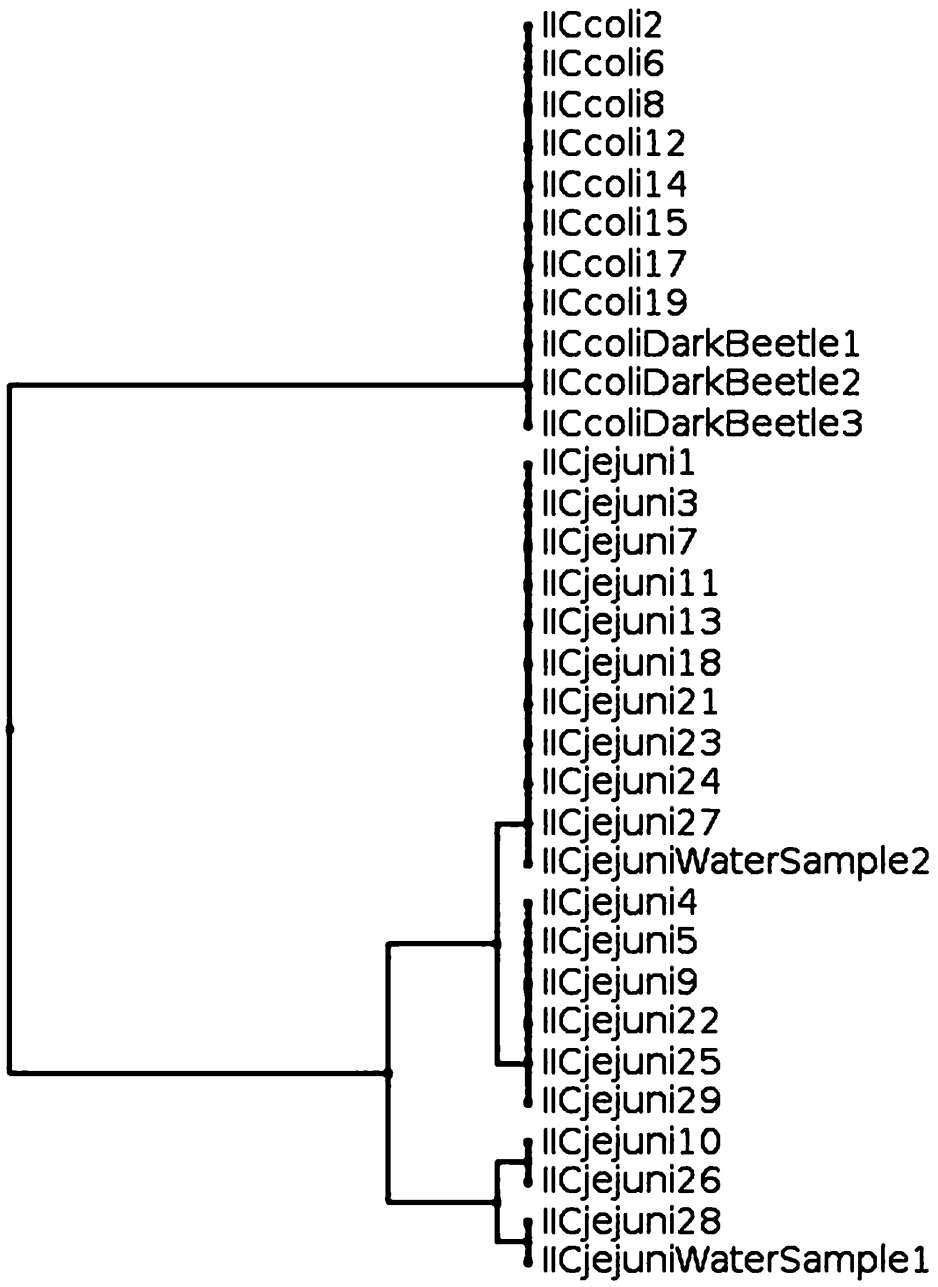
Dendrogram based on restriction profiles of *fla*A gene of 21 *C. jejuni* (19 from cloacal swabs and 2 from drinking water) and 11 *C. coli* (8 from cloacal swabs and 3 from dark beetles) isolated from farm II

**Fig. 4:**
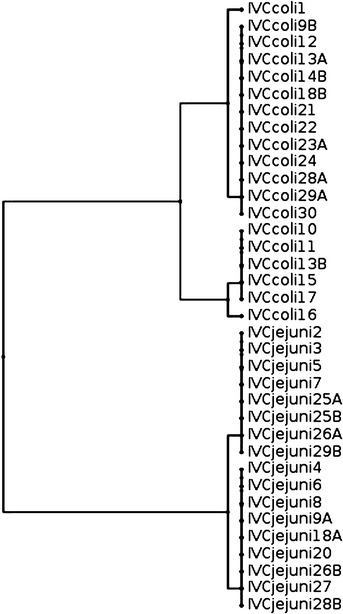
Dendrogram based on restriction profiles of *fla*A gene of 17 *C. jejuni* and 19 *C. coli* isolated from cloacal swabs in farm IV

**Fig. 5:**
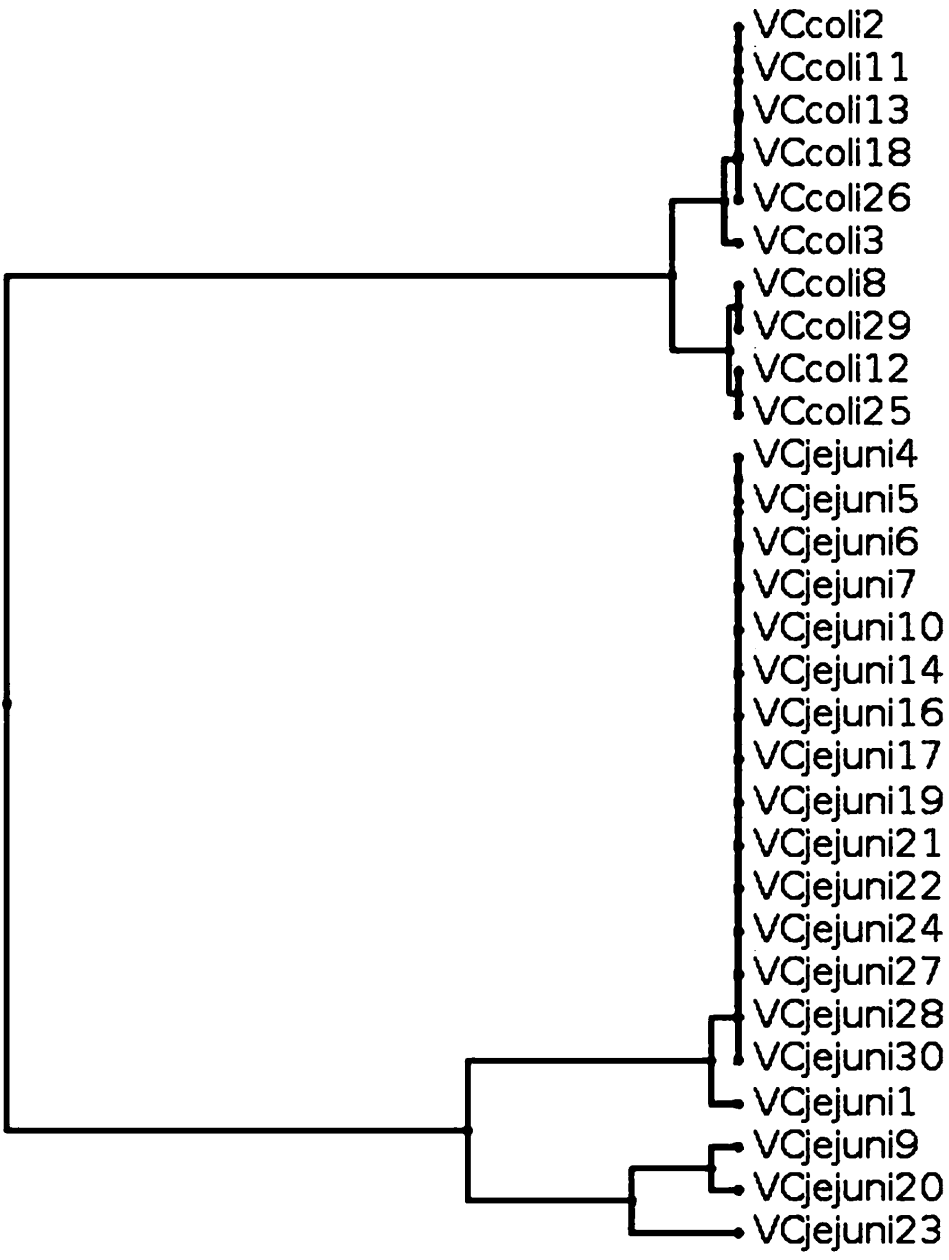
Dendrogram based on restriction profiles of *fla*A gene of 20 *C. jejuni* and 10 *C. coli* isolated from cloacal swabs in farm V

The genotypes of *Campylobacter* spp. isolated from 5 examined turkey farms either cloacal swabs or drinking water and darkling beetles by *fla*A-RFLP revealed 24 different genotypes. The relatedness and genetic diversity of genotypes was presented in Fig. 1. High genetic diversity was shown in the farms I, II, IV and V (Figs. 2, 3, 4, 5). While, in farm III only one genotype was found among *C. jejuni* and 2 genotypes of *C. coli* isolates.

Two different species *C. jejuni* and *C. coli* were isolated from one bird in farms III and IV.

In farm I the genotype of *C. coli* isolated from drinking water was identical with that of two isolates recovered from cloacal swabs (Fig. 2). In farm II two different genotypes were detected among *C. jejuni* isolated from drinking water which were identical with other isolates originated from cloacal swabs from turkey in the same farm. Three *C. coli* isolated from darkling beetles in farm II were similar and having same genotype which was identical with all 8 *C. coli* isolated from cloacal swabs from the same farm (Fig. 3).

### Effect of environmental parameters on occurrence of *Campylobacter*

No marked effects of the measured environmental parameters on the prevalence of *Campylobacter* spp. as temperature and atmosphere were found (Table 1). When the temperature ranged from 15.4 to 23 °C and the level of CO_2_ varied from 350 to 1400 ppm the *Campylobacter* prevalence was 90–100 % independent from both parameters. Similarly, prevalence of *Campylobacter* was 100 % when the RH ranged from 56.6 to 74.8 % and the ammonia concentration was between 0 and 22 ppm (Table 1).

## Discussion

There was little published information about the presence of *Campylobacter* spp. in organic turkey flocks. The results of the presented study on 5 turkey farms indicate that *Campylobacter* spp. seem to be highly prevalent in organic turkey production in Germany. 90–100 % of all cloacal swab samples from 150 tested samples on the 5 farms were *Campylobacter* positive. This finding is in general terms consistent with previous studies, which found prevalence of *Campylobacter* spp. infections in organic turkey operation ranging between 6 and 100 % [22]. The results are even more in accordance with studies on broilers from France which showed that 85.7 % of faecal samples from one flock of chickens raised in a free-range system were *Campylobacter* positive [27] and from Denmark where 100 % of the investigated cloacal samples from organic broiler flocks were *Campylobacter* positive [18]. The reason for the high prevalence in organic production systems can surely be explained by the permanent access of the free-range birds to the outdoor areas, where, they easily can close contact to wild birds and theirs faeces as well as to soil and rain water. Also infectious agents transmitted by air can come more easily in contact with free range birds than housed birds. The higher risk for free range birds compared to birds reared under conventional conditions [8] is documented in several studies indicating that open environment exposure has to be considered as additional risk factor for increasing the prevalence of *Campylobacter* in organic poultry production [19, 28].

However, the general production conditions have to be taken in account. A survey in Switzerland indicated that the *Campylobacter* prevalence in cloacal swabs of free-range birds (69 %) was only slightly higher (not significant) than that of conventionally reared broilers (50 %) [29]. However, the samples collected from the litter showed that the presence of a genotype in the environment of the birds does not implement that also the birds are infected [11].

In this study, *C. coli* and *C. jejuni* isolates were comparable and there were only slight differences. *C. coli* was the predominant species isolated from the organic turkey flocks with an overall prevalence of 54.3 % of all *Campylobacter* isolates. This result is in contrast to earlier studies performed in both organic turkey and broiler flocks, where *C. jejuni* was the highly prevalent species with 66 and 72 %, respectively [22, 30]. However, the result is in agreement with findings of Smith et al. [31] who revealed that 80–90 % of isolates colonizing commercial turkey flocks were *C. coli*. Similar results were recently reported by Kashoma et al. [32] who found that 72.3 % of all *Campylobacter* isolates in commercial turkey flocks were confirmed as *C. coli*.

Molecular typing with *fla*A-RFLP considered as differentiation tool for *Campylobacter* [33]. From 98 % of flocks testing positive, 10 birds (6.67 %) harboured both *C. jejuni* and *C. coli*. This result was supported by previous reports which found a mixture of both *Campylobacter* spp. in one bird [17, 34, 35].

*fla*A-RFLP assay results in this study showed that single turkey farms can harbour more than one genotype in one production cycle (4 types of *C. coli* in farm IV, V and 5 genotypes of *C. jejuni* in farm V). This finding was in accordance with previous study [36].

Isolation of *Campylobacter* spp. from the environment is generally poor as observed in this study, it may be due to numerous ambient stressors such as low temperature, dryness, radiation and nutrition competition which can have a negative effect on the viability of *Campylobacter* spp. as mentioned before [37]. Enrichment in Bolton broth was very important in this study to recover *Campylobacter* in the water from drinkers and darkling beetles. Despite of enrichment there was no *Campylobacter* isolated from water either from a farm owned well or as municipal water which in agreement with previous studies [38, 39]. Moreover, other studies concluded that water considered as a primary risk factor for occurrence and spreading of *Campylobacter* infection within the flock [40, 41]. Furthermore, in a previous longitudinal study, *Campylobacter* DNA could be detected from drinkers after 6 days of stocking and before detection of infection in pullets [17]. On the other hand, studies considered that drinking water unlikely to be responsible on introduction of *Campylobacter* infection into poultry farms [42, 43].

Similar to the debate on the drinking water as a vector for *Campylobacter* transmission the role of darkling beetles is discussed. Direction of infection is not clear whether the beetles are carrying *Campylobacter* first and transmit it to the birds [13] or the birds excrete *Campylobacter* which were taken up by the beetles, acting as alternate vectors and source of infection [39, 44]. Even a single exposure of chicks to contaminated insects may be sufficient for colonization of the bird intestines as observed in a previous study [45]. These previously mentioned explanation support our findings as we detect the *Campylobacter* in beetles in one flock despite all flocks tested positive with high prevalence. The role of the contaminated beetles in *Campylobacter* transmission was discussed in previous studies [12, 14, 46] as some of these reports proved their role while others deny due to the short duration (few days) of bacterial carriage by the beetles.

In this study the molecular typing of isolated *Campylobacter* showed identity between genotypes detected in flocks and environmental samples which supported previous studies [10, 47].

The significance of air quality (ammonia and CO_2_ level) with *Campylobacter* occurrence in birds has not been frequently addressed. In this study, we did not found any influence of air quality on *Campylobacter* prevalence in examined flocks.

## Conclusions

The results of this study provided new information about the *Campylobacter* prevalence in German organic turkey production and pointed out some potential sources of *Campylobacter* spreading for this kind of rearing system. This study showed that the water and darkling beetles considered as risk factor for presence of *Campylobacter* in organic turkey farm that should be taken into account during cleaning and disinfection of farm. Moreover, an influence of air quality on *Campylobacter* prevalence was not found in the sporadic and short time measurement and need further investigation.

## Additional files

10.1186/s13099-016-0108-2 Bird and environmental samples identification with conventional and molecular method and flock description data.
10.1186/s13099-016-0108-2 The within flock prevalence of *Campylobacter* isolated from 30 cloacal swabs in 5 different organic turkey flocks and *C. jejuni*, *C. coli* genotypes and *Campylobacter* positive environmental samples (water tank, water at birds and darkling beetles) in 5 different organic turkey flocks.

## Abbreviations

spp.: species
NH_3_: ammonia
CO_2_: carbon dioxide
RH: relative humidity
MFT: membrane filtration technique
mCCDA: modified Charcoal Cefoperazone Desoxycholate Agar
mPCR: multiplex PCR
RFLP: restriction fragment length polymorphism
