# Supplementary material for: Prevalence, genotyping and risk factors of thermophilic Campylobacter spreading in organic turkey farms in Germany
Source: Gut Pathog. 2016 Jun 2;8:28. doi: 10.1186/s13099-016-0108-2 (PMC4890334; doi:10.1186/s13099-016-0108-2)
Supplement: Supplementary file 1 — 10.1186/s13099-016-0108-2 Bird and environmental samples identification with conventional and molecular method and flock description data. [file 13099_2016_108_MOESM1_ESM.pdf]

Farm 1

Sample ID Nr.	Molecular mPCR-method	Conventional method	Cryobank Nr.
31512-ET1a-CS1	*C. coli*	*C. coli*	230712CS0134
31512-ET1a-CS2	*C. coli*	*C. coli*	230712CS0135
31512-ET1a-CS3	*C. coli*	*C. coli*	230712CS0136
31512-ET1a-CS4	*C. coli*	*C. coli*	230712CS0137
31512-ET1a-CS5	*C. coli*	*C. coli*	230712CS0138
31512-ET1a-CS6	*C. coli*	*C. coli*	230712CS0139
31512-ET1a-CS7	*C. coli*	*C. coli*	230712CS0140
31512-ET1a-CS8	*C. coli*	*C. coli*	230712CS0141
31512-ET1a-CS9	*C. jejuni*	*C. jejuni*	230712CS0142
31512-ET1a-CS10	*C. coli*	*C. coli*	230712CS0143
31512-ET1a-CS11	*C. coli*	*C. coli*	230712CS0144
31512-ET1a-CS12	*C. jejuni*	*C. jejuni*	230712CS0145
31512-ET1a-CS13	*C. coli*	*C. coli*	230712CS0146
31512-ET1a-CS14	*C. coli*	*C. coli*	230712CS0147
31512-ET1a-CS15	*C. jejuni*	*C. jejuni*	230712CS0148
31512-ET1a-CS16	*C. coli*	*C. coli*	230712CS0149
31512-ET1a-CS17	*C. coli*	*C. coli*	230712CS0150
31512-ET1a-CS18	*C. coli*	*C. coli*	230712CS0151
31512-ET1a-CS19	*C. jejuni*	*C. jejuni*	230712CS0152
31512-ET1a-CS20	*C. coli*	*C. coli*	230712CS0153
31512-ET1a-CS21	*C. jejuni*	*C. jejuni*	230712CS0154
31512-ET1a-CS22	*C. coli*	*C. coli*	230712CS0155
31512-ET1a-CS23	*C. jejuni*	*C. jejuni*	230712CS0156
31512-ET1a-CS24	*C. coli*	*C. coli*	230712CS0157
31512-ET1a-CS25	*C. coli*	*C. coli*	230712CS0158
31512-ET1a-CS26	*C. jejuni*	*C.jejuni*	230712CS0159
31512-ET1a-CS27	*C. coli*	*C. coli*	230712CS0160
31512-ET1a-CS28	*C. jejuni*	*C. jejuni*	230712CS0161
31512-ET1a-CS29	*C. coli*	*C. coli*	230712CS0162
31512-ET1a-CS30	*C. coli*	*C. coli*	230712CS0163
31512-ET1a-WS1	*C. coli*	*C. coli*	230712CS0164

Farm1

Item	Flock Data
Sampling time	31/05/2012
Birds no.	1003 (at start was 1030)
Birds age (w)	8 weeks
Breed	Kelly BBB
Temp. C	15,8 C
RH%	63.6
Ammonia (ppm)	1 ppm
Co2 (ppm)	500 ppm
Water supply type	Tap water
Drinkers type	7 cups+ 15 pendolus
positive clowacl swa	30
positive water samp	1
beetles sample	negative
C. jejuni positive	8 Cloacal Swabs
C. coli positive	22 Cloacal Swabs+1Water Sample
C.jejuni genotypes	3
C. coli genotypes	2

Farm 2

**Sample ID Nr.**	**Molecular mPCR-method**	**Conventional method**	**Cryobank Nr.**
7612-ET2a-CS1	*C. jejuni*	*C. jejuni*	130712CS0102
7612-ET2a-CS2	*C. coli*	*C. coli*	130712CS0103
7612-ET2a-CS3	*C. jejuni*	*C. jejuni*	130712CS0104
7612-ET2a-CS4	*C. jejuni*	*C. jejuni*	130712CS0105
7612-ET2a-CS5	*C. jejuni*	*C. jejuni*	130712CS0106
7612-ET2a-CS6	*C. coli*	*C. coli*	130712CS0107
7612-ET2a-CS7	*C. jejuni*	*C. jejuni*	130712CS0108
7612-ET2a-CS8	*C. coli*	*C. coli*	130712CS0109
7612-ET2a-CS9	*C. jejuni*	*C. jejuni*	130712CS0110
7612-ET2a-CS10	*C. jejuni*	*C. jejuni*	130712CS0111
7612-ET2a-CS11	*C. jejuni*	*C. jejuni*	130712CS0112
7612-ET2a-CS12	*C. coli*	*C. coli*	130712CS0113
7612-ET2a-CS13	*C. jejuni*	*C. jejuni*	130712CS0114
7612-ET2a-CS14	*C. coli*	*C. coli*	130712CS0115
7612-ET2a-CS15	*C. coli*	*C. coli*	130712CS0116
7612-ET2a-CS17	*C. coli*	*C. coli*	130712CS0117
7612-ET2a-CS18	*C. jejuni*	*C. jejuni*	130712CS0118
7612-ET2a-CS19	*C. coli*	*C. coli*	130712CS0119
7612-ET2a-CS21	*C. jejuni*	*C. jejuni*	130712CS0120
7612-ET2a-CS22	*C. jejuni*	*C. jejuni*	130712CS0121
7612-ET2a-CS23	*C. jejuni*	*C. jejuni*	130712CS0122
7612-ET2a-CS24	*C. jejuni*	*C. jejuni*	130712CS0123
7612-ET2a-CS25	*C. jejuni*	*C. jejuni*	130712CS0124
7612-ET2a-CS26	*C. jejuni*	*C. jejuni*	130712CS0125
7612-ET2a-CS27	*C. jejuni*	*C. jejuni*	130712CS0126
7612-ET2a-CS28	*C. jejuni*	*C. jejuni*	130712CS0127
7612-ET2a-CS29	*C. jejuni*	*C. jejuni*	130712CS0128
7612-ET2a-WS1	*C. jejuni*	*C. jejuni*	130712CS0129
7612-ET2a-WS2	*C. jejuni*	*C. jejuni*	130712CS0130
7612-ET2a-DB1	*C. coli*	*C. coli*	130712CS0131
7612-ET2a-DB2	*C. coli*	*C. coli*	130712CS0132
7612-ET2a-DB3	*C. coli*	*C. coli*	130712CS0133

Farm 2

Item	Flock Data
Sampling time	07/06/2012
Hatching day	10/04/2012
Birds no.	2000
Birds age (w)	8 weeks
Breed	B.U.T. 6
Temp. C	23.1
RH%	64.2
Ammonia (ppm)	14
Co2 (ppm)	1400
Water supply type	Tap water
Drinkers type	17 cups
positive clowacl swa	27
positive water samp	2 (C. jejuni)
positive beetles sam	3 (C. coli)
C. jejuni positive	21 (19 Cloacal Swabs + 2 Water Samples)
C. coli positive	11 (8 Cloacal Swabs + 3 Darkling Beetls)
C.jejuni genotypes	4
C. coli genotypes	1

Farm 3

Sample ID Nr.	Molecular mPCR-method	Conventional method	Cryobank Nr.
14612-ET3a-CS1	*C. coli*	*C. coli*	051012CS0213
14612-ET3a-CS2	*C. coli*	*C. coli*	051012CS0214
14612-ET3a-CS3	*C. coli*	*C. coli*	051012CS0215
14612-ET3a-CS4	*C. coli*	*C. coli*	051012CS0216
14612-ET3a-CS5	*C. jejuni*	*C.jejuni jejeuni 1*	051012CS0217
14612-ET3a-CS6	*C. coli*	*C. coli*	051012CS0218
14612-ET3a-CS7	*C. coli*	*C. coli*	051012CS0219
14612-ET3a-CS8	*C. coli*	*C. coli*	051012CS0220
14612-ET3a-CS9	*C. coli*	*C. coli*	051012CS0221
14612-ET3a-CS10	*C. coli*	*C. coli*	051012CS0222
14612-ET3a-CS11	*C. coli*	*C. coli*	051012CS0223
14612-ET3a-CS12	*C. coli*	*C. coli*	051012CS0224
14612-ET3a-CS13	*C. coli*	*C. coli*	051012CS0225
14612-ET3a-CS14-1	*C. coli*	*C. coli*	051012CS0226
14612-ET3a-CS14-2	*C. jejuni*	*C.jejuni jejeuni 2*	051012CS0227
14612-ET3a-CS15	*C. coli*	*C. coli*	051012CS0228
14612-ET3a-CS16	*C. coli*	*C. coli*	051012CS0228
14612-ET3a-CS17	*C. coli*	*C. coli*	051012CS0229
14612-ET3a-CS18	*C. coli*	*C. coli*	051012CS0230
14612-ET3a-CS19	*C. coli*	*C. coli*	051012CS0231
14612-ET3a-CS20	*C. coli*	*C. coli*	051012CS0232
14612-ET3a-CS21-1	*C. coli*	*C. coli*	051012CS0233
14612-ET3a-CS21-2	*C. jejuni*	*C.jejuni jejeuni 2*	051012CS0234
14612-ET3a-CS22	*C. coli*	*C. coli*	051012CS0235
14612-ET3a-CS23-1	*C. coli*	*C. coli*	051012CS0236
14612-ET3a-CS23-2	*C. coli*	*C.jejuni jejeuni 1*	051012CS0237
14612-ET3a-CS24-1	*C. coli*	*C. coli*	051012CS0238
14612-ET3a-CS24-2	*C. jejuni*	*C.jejuni jejeuni 1*	051012CS0239
14612-ET3a-CS25-1	*C. coli*	*C. coli*	051012CS0240
14612-ET3a-CS25-2	*C. jejuni*	*C.jejuni jejeuni 1*	051012CS0241
14612-ET3a-CS26	*C. coli*	*C. coli*	051012CS0242
14612-ET3a-CS27	*C. coli*	*C. coli*	051012CS0243
14612-ET3a-CS28	*C. coli*	*C. coli*	051012CS0244
14612-ET3a-CS29	*C. coli*	*C. coli*	051012CS0245
14612-ET3a-CS30	*C. coli*	*C. coli*	051012CS0246

Farm 3

Item	Flock Data
Sampling time	14/06/2012
Hatching day	15/05/2012
Birds no.	1400
Birds age (w)	4
Breed	B.U.T. 6 British united turkey
Temp. C	21.30
RH%	74.8
Ammonia (ppm)	22
Co2 (ppm)	800
Water supply type	well water
Drinkers type	6 cups+ 4 drinking trough
positive clowacl swab	35
water samples	negative
beetles sample	negative
C. jejuni positive	5 Cloacal Swabs
C. coli positive	30 Cloacal Swabs
C.jejuni genotypes	1
C. coli genotypes	2
Hatching day	15/05/2012

Farm 4

**Sample ID Nr.**	**Molecular mPCR-method**	**Conventional method**	**Cryobank Nr.**
12712-ET6a-CS1	*C. coli*	*C.jejuni jejuni 1*	070912CS0167
12712-ET6a-CS2	*C. jejuni*	*C.jejuni jejuni 1*	070912CS0168
12712-ET6a-CS3	*C. jejuni*	*C.jejuni jejuni 1*	070912CS0169
12712-ET6a-CS4	*C. jejuni*	*C.jejuni jejuni 2*	070912CS0170
12712-ET6a-CS5	*C. jejuni*	*C.jejuni jejuni 1*	070912CS0171
12712-ET6a-CS6	*C. jejuni*	*C.jejuni jejuni 2*	070912CS0172
12712-ET6a-CS7	*C. jejuni*	*C.jejuni jejuni 1*	070912CS0173
12712-ET6a-CS8	*C. jejuni*	*C.jejuni jejuni 2*	070912CS0174
12712-ET6a-CS9-1	*C. jejuni*	*C.jejuni jejuni 1*	070912CS0175
12712-ET6a-CS9-2	*C. coli*	*C. coli*	070912CS0176
12712-ET6a-CS10	*C. coli*	*C. coli*	070912CS0177
12712-ET6a-CS11	*C. coli*	*C. coli*	070912CS0178
12712-ET6a-CS12	*C. coli*	*C.coli*	070912CS0179
12712-ET6a-CS13-1	*C. coli*	*C.coli*	070912CS0180
12712-ET6a-CS13-2	*C. coli*	*C. coli*	070912CS0181
12712-ET6a-CS14-2	*C. coli*	*C.jejuni jejuni 1*	070912CS0183
12712-ET6a-CS15	*C. coli*	*C.coli*	070912CS0184
12712-ET6a-CS16	*C. coli*	*C.coli*	070912CS0185
12712-ET6a-CS17	*C. coli*	*C.coli*	070912CS0186
12712-ET6a-CS18-1	*C. jejuni*	*C.coli*	070912CS0187
12712-ET6a-CS18-2	*C. coli*	*C.coli*	070912CS0188
12712-ET6a-CS20	*C. jejuni*	*C.jejuni jejuni 1*	070912CS0189
12712-ET6a-CS21	*C. coli*	*C.coli*	070912CS0190
12712-ET6a-CS22	*C. coli*	*C.coli*	070912CS0191
12712-ET6a-CS23-1	*C. coli*	*C.coli*	070912CS0192
12712-ET6a-CS24	*C. coli*	*C.coli*	070912CS0193
12712-ET6a-CS25-1	*C. jejuni*	*C.jejuni jejuni 1*	070912CS0194
12712-ET6a-CS25-2	*C. jejuni*	*C.jejuni jejuni 1*	070912CS0195
12712-ET6a-CS26-1	*C. jejuni*	*C.jejuni jejuni 1*	070912CS0196
12712-ET6a-CS26-2	*C. jejuni*	*C.jejuni jejuni 1*	070912CS0197
12712-ET6a-CS27	*C. jejuni*	*C.jejuni jejuni 2*	070912CS0198
12712-ET6a-CS28-1	*C. coli*	*C.coli*	070912CS0199
12712-ET6a-CS28-2	*C. jejuni*	*C.jejuni jejuni 2*	070912CS0200
12712-ET6a-CS29-1	*C. coli*	*C.coli*	070912CS0201
12712-ET6a-CS29-2	*C. jejuni*	*C.jejuni jejuni 1*	070912CS0202
12712-ET6a-CS30	*C. coli*	*C.coli*	070912CS0203

Farm 4

Item	Flock Data
Sampling time	12/07/2012
Birds no.	1100
Birds age (w)	8 weeks
Breed	Kelly BBB
Temp. C	21.80
RH%	58.3
Ammonia (ppm)	1 ppm
Co2 (ppm)	350 ppm
Water supply type	Tap water
Drinkers type	Penduous
positive clowacl swab	38
water samples	negative
beetles sample	negative
C. jejuni positive	17
C. coli positive	19
C.jejuni genotypes	2
C. coli genotypes	4

Farm 5

**Sample ID Nr.**	**Molecular mPCR-method**	**Conventional method**	**Cryobank Nr.**
6912-ET7-CS1	*C. jejuni*	*C.jejuni jejeuni 1*	051012CS0243
6912-ET7-CS2	*C. coli*	*C. coli*	051012CS0244
6912-ET7-CS3	*C. coli*	*C. coli*	051012CS0245
6912-ET7-CS4	*C. jejuni*	*C.jejuni jejeuni 1*	051012CS0246
6912-ET7-CS5	*C. jejuni*	*C.jejuni jejeuni 1*	051012CS0247
6912-ET7-CS6	*C. jejuni*	*C.jejuni jejeuni 1*	051012CS0248
6912-ET7-CS7	*C. jejuni*	*C.jejuni jejeuni 1*	051012CS0249
6912-ET7-CS8	*C. coli*	*C. coli*	051012CS0250
6912-ET7-CS9	*C. jejuni*	*C.jejuni jejeuni 1*	051012CS0251
6912-ET7-CS10	*C. jejuni*	*C.jejuni jejeuni 1*	051012CS0252
6912-ET7-CS11	*C. coli*	*C.jejuni jejeuni 1*	051012CS0253
6912-ET7-CS12	*C. coli*	*C.jejuni doylei*	051012CS0254
6912-ET7-CS13	*C. coli*	*C. coli*	051012CS0255
6912-ET7-CS14	*C. jejuni*	*C.jejuni jejeuni 1*	051012CS0256
6912-ET7-CS15	*C. jejuni*	*C.jejuni jejeuni 1*	051012CS0257
6912-ET7-CS16	*C. jejuni*	*C.jejuni jejeuni 1*	051012CS0258
6912-ET7-CS17	*C. jejuni*	*C.jejuni jejeuni 1*	051012CS0259
6912-ET7-CS18	*C. coli*	*C. coli*	051012CS0260
6912-ET7-CS19	*C. jejuni*	*C.jejuni doylei*	051012CS0261
6912-ET7-CS20	*C. jejuni*	*C.jejuni jejeuni 1*	051012CS0262
6912-ET7-CS21	*C. jejuni*	*C.jejuni jejeuni 1*	051012CS0263
6912-ET7-CS22	*C. jejuni*	*C.jejuni jejeuni 1*	051012CS0264
6912-ET7-CS23	*C. jejuni*	*C.jejuni jejeuni 1*	051012CS0265
6912-ET7-CS24	*C. jejuni*	*C.jejuni jejeuni 1*	051012CS0266
6912-ET7-CS25	*C. coli*	*C.jejuni jejeuni 1*	051012CS0267
6912-ET7-CS26	*C. coli*	*C.jejuni doylei*	051012CS0268
6912-ET7-CS27	*C. jejuni*	*C. jejuni*	051012CS0269
6912-ET7-CS28	*C. jejuni*	*C.jejuni jejeuni 1*	051012CS0270
6912-ET7-CS29	*C. coli*	*C.jejuni jejeuni 1*	051012CS0271
6912-ET7-CS30	*C. jejuni*	*C. coli*	051012CS0272

Farm 5

Items	Flock Data
Sampling time	06/09/2012
Birds no.	1500
Birds age (w)	6
Breed	B.U.T. 6 British united turkey
Temp. C	15.4
RH%	56.6
Ammonia (ppm)	0
Co2 (ppm)	400
Water supply type	Tap water
Drinkers type	16 cups
positive clowacl swab	30
water samples	negative
beetles sample	negative
C. jejuni positive	20
C. coli positive	10
C.jejuni genotypes	5
C. coli genotypes	4
