# Supplementary material for: Prevalence, genotyping and risk factors of thermophilic Campylobacter spreading in organic turkey farms in Germany
Source: Gut Pathog. 2016 Jun 2;8:28. doi: 10.1186/s13099-016-0108-2 (PMC4890334; doi:10.1186/s13099-016-0108-2)
Supplement: Supplementary file 2 — 10.1186/s13099-016-0108-2 The within flock prevalence of Campylobacter isolated from 30 cloacal swabs in 5 different organic turkey flocks and C. jejuni, C. coli genotypes and Campylobacter positive environmental samples (water tank, water at birds and darkling beetles) in 5 different organic turkey flocks. [file 13099_2016_108_MOESM2_ESM.pdf]

The within flock prevalence of Campylobacter isolated from 30 cloacal swabs in 5 different organic turkey flocks and C.jejuni, C. Coli genotyp

	Campylobacter (%) of positive	C. jejuni (%)of positive	C. coli (%) of positive	No. of C. Jejuni genotypes	No. of C. Coli genotypes
Flock 1 (I)	100	26.67	73.33	3	2
Flock 2 (II)	90	70.37	29.63	4	1
Flock 3 (III)	100	14.29	85.71	1	2
Flock 4 (VI)	100	47.22	52.78	2	4
Flock 5 (V)	100	66.67	33.33	5	4
Total	98.00	45.04	54.96	15	13

Campylobacter positive from environmental samples (water tank, water at birds and darkling beetles) in 5 different organic turkey flocks

		Water tank	Drinkers water sample			Darkling beetels (n=5)
		500 ml	1000 ml	500 ml	1000 ml	
Flock 1 (I)	with enrichement	-	-	-	+	-
	without enrichement	-	-	-	-	-
Flock 2 (II)	with enrichement	-	-	+	+	3
	without enrichement	-	-	-	-	-
Flock 3 (III)	with enrichement	-	-	-	-	-
	without enrichement	-	-	-	-	-
Flock 6 (VI)	with enrichement	-	-	-	-	-
	without enrichement	-	-	-	-	-
Flock 7 (V)	with enrichement	-	-	-	-	-
	without enrichement	-	-	-	-	-
					BWS C. Coli	
				BWS C. Jejuni	BWS C. Jejuni	DB C. Coli

es
